# The Paralysis of Osteo-Arthritis

**Published:** 1895-06

**Authors:** Henry Waldo

**Affiliations:** Physician to the Bristol Royal Infirmary


### THE PARALYSIS OF OSTEO-ARTHRITIS.

BY

HENRY WALDO, M.D. Aberd., M.R.C.P. Lond.,

*Physician to the Bristol Royal Infirmary.*

MANY cases of osteo-arthritis are associated with loss of power, and often with some slight sensory affection. A sense of weakness and fatigue is nearly always present, but the paresis is much obscured by the pain.

The loss of power in this condition seems to depend partly upon the pain in the joint which movement causes, and which is nearly always attended with stiffness, but the loss of power mostly depends upon the great wasting of muscles which takes place. There is very seldom any sensory affection besides the pain, but in some cases there is tingling in the skin and hyperæsthesia. It is said that areas of anæsthesia are sometimes found; but it is probable that, in such cases, inflammation has extended to a nerve in the vicinity of the joint. The muscles that waste are chiefly those which extend the affected joint. It, however, sometimes involves the flexors as well as the extensors, and rarely muscles of the limb that are near but do not move the affected joint. The atrophied muscles are nearly always on the proximal side of the affected joint. When wasting occurs on the distal side it is often owing to the inflammation spreading from the joint to a nerve, and thus producing distant wasting. The atrophy seems to be especially rapid when there is much pain in the joint. However long the affected muscle (on the proximal side of the affected joint) may be, the wasting involves the whole length of it—the part of the muscle at a distance from the joint wastes as much as the part near the joint. The wasting increases for some weeks, then becomes stationary, and continues as long as the joint-disease lasts. If the joint recovers, the muscles in most cases slowly regain their normal size. The electrical irritability of the atrophied muscles is usually normal, but it is often slightly lessened, equally to faradaism and voltaism. It is curious that this atrophy of muscles is generally accompanied by a distinct increase in myotatic irritability. The knee-jerk is excessive if the thigh-muscles are affected, and a rectus-clonus can sometimes be obtained. A foot-clonus may be present when the ankle joint is affected. In some cases there is a slight rigidity of the affected muscles. Rarely some contracture occurs in the opponents of the atrophied muscles. The plantar skin-reflex is most markedly exaggerated in some cases. The legs fly up and often cause much pain, and the patients beg not to have the skin of the sole touched again.

At the present time the profession seems inclined to regard the nervous system as the origin of this disease. But there is very little proof of this. The pathology suggested by Paget in cases of arthritic muscular atrophy is, "a reflex atrophy, due to the disturbance of some nutritive nervous centre, irritated by the painful state of the sensitive nerve-fibres." It is thought by others that the wasting may be due to alterations in the nutrition of the ganglion cells of the cord. If this is the true explanation, it must be that form of wasting which Gowers calls "tonic atrophy," in which the ganglion cells are only partially affected. Other writers only assume that the wasting is produced, in some way, through the nervous system.

Folli discusses the pathology of osteo-arthritis (which he calls chronic polyarticular rheumatism) in respect to the nervous theory. "He reports in full three cases with the minute morbid anatomy. In two the clinical manifestations were quite typical, the hands presenting the characteristic deformity. The wrist, elbow, and shoulder-joints were also affected, but in decreasing degrees. Here lesions were found in the anterior horns—atrophic in the first case, but degenerative as well as atrophic in the second. The lesion became less marked in passing down towards the periphery through anterior nerve-roots, nerves, and muscles. In the third case the disease was most marked in the shoulder-joints, gradually diminishing downwards, so that the hands were but slightly affected. Here the morbid changes were but faintly marked in the nervous system, whereas the muscles and articulations were profoundly altered. The more minute lesions in the first two cases consisted in a diminution in the number of cells in the anterior horns, with a disappearance of their processes and increased deposit of pigment. There was a rarefaction of the neuroglia, more marked towards the inner parts and at the junction of the two horns. Where the neuroglia was most attenuated the alteration in the cells was greatest. The nerve-roots and nerves were also atrophied. Folli thinks that the lesions in the cord were in all probability primary, because the changes were so pronounced there, and because disease in the cord can cause arthropathies. He gives details of a fourth case of this disease, death taking place from tuberculosis. Here cavities were present in the grey matter, and they corresponded to places where, in the other cases, the neuroglia was most thinned and the cells most atrophied. Folli does not believe that the lesions could be attributed to disease of the limbs. . . . All cases of arthritis deformans cannot be thus explained. In Case 3 the lesions in the spinal cord were too slight to attach importance to." [1]

[1] *Policlinico*, Dec., 1894, quoted in *Brit. M. J.*, 1895, vol. i., Epitome, p. 17.

It must not be overlooked that osteo-arthritis, with its attendant atrophy, appears to be caused by slight repeated irritation to joints; that it is often a degenerative or senile change; that overwork may account for it; and that, in many cases, there is a distinct traumatic origin.

Again, it is undoubtedly more common in damp countries—for instance, it is much more often met with in Ireland than in England or Scotland. In gouty joints the reverse of this occurs.

It is also more frequent in agricultural labourers than in town folk—although, taken as a whole, it occurs much oftener in women. Pathological anatomy throws no light on the mechanism by which the wasting is produced. The only constant condition in these cases that can be regarded as the cause of the wasting is the simple fact of the joint disease.

Disuse will not explain the atrophy—first, because its influence in causing wasting is trifling and tardy; and, secondly, because other muscles of a limb, equally disused, present no wasting.

I have noticed that these patients are rather liable to what are called bilious attacks, which somewhat resemble the gastric crises of tabes dorsalis, but are not so severe.

A cautious prognosis should be given, for, even if the joint gets well, the wasting often lasts for a long time; but, even if slight wasting is persistent, normal power is usually recovered.

The paresis does not at all correspond to cases of peripheral neuritis; for although the extensors are affected most in both conditions, and ankle-drop and wrist-drop exist, other signs do not correspond. The knee-jerk in peripheral neuritis is absent, excepting occasionally in the initiative stage, when it may be excessive for a time, as first pointed out by Ross. Ankle-clonus is out of the question. There is, as a rule, anæsthesia of the skin and tenderness of the calf muscles, as well as of nerve-trunks. The electrical reactions are those of degeneration. The muscular wasting being attended with increased myotatic irritability in osteo-arthritis, is very singular and difficult to explain.

The local treatment of the paresis depends upon the nutrition of the muscles, which is best carried out by electrical stimulation and gentle rubbing. It should be delayed until the joint-pain has subsided.

The general treatment should consist of fairly good living, with animal food two or three times a day. Some kind of stimulant with food—perhaps malt liquor—will do good. Cod-liver oil and arsenic, with the occasional daily hypodermic injection of nitrate of strychnia, appear to be of more benefit than any other drugs. Exercise, short of producing fatigue, should be persevered in, seeing that all the affected joints are judiciously moved. I think at the present day we are a little inclined to feed these helpless patients too well. It must be remembered that they are able at best to take very little exercise, and this associated with a too liberal dietary is liable to induce another disease—namely, gout. No doubt osteo-arthritis and deposits of urate of soda are sometimes present in the same patient.

